# Supplementary material for: Algo-Functional Indexes and Spatiotemporal Parameters of Gait after Sacroiliac Joint Arthrodesis
Source: J Clin Med. 2020 Sep 4;9(9):2860. doi: 10.3390/jcm9092860 (PMC7563510; doi:10.3390/jcm9092860)
Supplement: Supplementary file 1 [file jcm-09-02860-s001.pdf]

Supplementary Table 1. Italian version of the Majeed questionnaire.
Scoring numbers are reported in italic.

**Sezione 1. Dolore** (30 punti)		
☐	Severo, continuo e presente a riposo	*0*
☐	Severo durante l'attività	*10*
☐	Tollerabile ma limita l'attività	*15*
☐	Presente per attività moderata, assente a riposo	*20*
☐	Moderato, intermittente, non limita l'attività normale	*25*
☐	Lieve, occasionale o assente	*30*
**Sezione 2. Attività lavorativa** (20 punti)		
☐	Nessuna occupazione regolare	*0*
☐	Lavoro leggero	*8*
☐	Cambio di lavoro	*12*
☐	Stesso lavoro, riduzione del rendimento	*16*
☐	Stesso lavoro, stesso rendimento	*20*
**Sezione 3. Stare seduto/a** (10 punti)		
☐	Causa dolore	*0*
☐	Causa dolore se prolungato o scomodo	*6*
☐	Non confortevole	*8*
☐	Senza limitazioni	*10*
**Sezione 4. Rapporto sessuale** (4 punti)		
☐	Causa dolore	*0*
☐	Causa dolore se prolungato o scomodo	*6*
☐	Non confortevole	*8*
☐	Senza limitazioni	*10*
**Sezione 5. Camminare con ausili** (12 punti)		
☐	Allettato o quasi	*0*
☐	Carrozzina	*4*
☐	Due stampelle	*6*
☐	Due bastoni	*8*
☐	Un bastone	*10*
☐	No ausili	*12*
**Sezione 6. Cammino autonomo** (12 punti)		
☐	Impossibile o quasi	*0*
☐	Strisciante a piccoli passi	*4*
☐	Zoppia severa	*6*
☐	Zoppia moderata	*8*
☐	Zoppia lieve	*10*
☐	Normale	*12*
**Sezione 7. Distanza del cammino** (12 punti)		
☐	Allettamento o percorso di pochi metri	*0*
☐	Tempo e distanze molti limitati	*4*
☐	Limitato con bastoni, difficile senza possibilità di mantenimento prolungato della stazione eretta	*6*
☐	Un'ora con un bastone, limitato senza	*8*
☐	Un'ora senza bastoni con lieve dolore o zoppia	*10*
☐	Normale per l'età e le condizioni generali	*12*

Supplementary Table 2. Italian version of the Majeed questionnaire.
Scoring numbers are reported in italic.

**ATTIVITÀ DELLA VITA QUOTIDIANA**<br>Riesce a:	*1 punto per ogni risposta SI*
Camminare 1,5 km	□ SI □ NO
Piegarsi sopra un lavandino	□ SI □ NO
Trasportare una borsa della spesa	□ SI □ NO
Rifare il letto	□ SI □ NO
Stare in auto	□ SI □ NO
Stare seduta/o per un periodo prolungato (ad esempio per guardare un film)	□ SI □ NO
Fare visita ad amici o parenti per una serata	□ SI □ NO
Stare in piedi per un'ora o più (per esempio ad aspettare in coda)	□ SI □ NO
Rastrellare le foglie o tagliare l'erba	□ SI □ NO
Sollevare bambini piccoli	□ SI □ NO
Spazzare il pavimento con una scopa	□ SI □ NO
Vestirsi senza assistenza	□ SI □ NO
Cucinare un pasto	□ SI □ NO
Svolgere attività ricreative leggere (esempio bowling, ballare)	□ SI □ NO
Svolgere attività ricreative vigorose (esempio corsa, tennis)	□ SI □ NO
Accovacciarsi	□ SI □ NO
Salire e scendere la scale normalmente (piede dopo piede)	□ SI □ NO
Salire e scendere le scale con adattamenti (uno scalino alla volta)	□ SI □ NO
Svolgere attività che richiedano di raggiungere qualcosa sopra la testa	□ SI □ NO
Dormire in maniera confortevole	□ SI □ NO
**PUNTEGGIO INDIVIDUALE**	
**Attività della vita quotidiana** *(20 punti) / 1 punto per ogni risposta SI*	
**Attività lavorativa**	
□ Nessuna variazione	*20*
□ A tempo pieno ma variata	*15*
□ Part time	*10*
□ Inabile	5
**Dolore**	
□ Assente, non significativo	*25*
□ (Necessita di) trattamento occasionale	*20*
□ (Necessita di) trattamento regolare	*15*
□ (Necessita di) ospedalizzazione/intervento	5
**Zoppia**	
□ No	*20*
□ Si	*15*
□ (Necessita di) ausili	*10*
□ Non deambulante	5
**Cura di sè**	
□ Nessun cambiamento	5
□ Cambiamento significativo	*0*
**Linea del dolore** *(indichi l'intensità del Suo dolore con una freccia sulla linea riportata)*	

10 *0*

0 **10**

Nessun dolore **Dolore insopportabile**
